# The association between parenting styles, maternal self-efficacy, and social and emotional adjustment among Arab preschool children

**DOI:** 10.1186/s41155-023-00252-4

**Published:** 2023-04-26

**Authors:** Qutaiba Agbaria, Fayez Mahamid

**Affiliations:** 1 Al-Qasemi College, Baqa al-Gharbiyye, Israel; 2 Psychology and Counseling Department, An-Najah National University, Nablus, Palestine

**Keywords:** Parenting styles, Maternal self-efficacy, Social-emotional adjustment, Palestinian preschool children

## Abstract

Parenting styles and parental self-efficacy are major factors that affect the overall adjustment of children. The current study examined parenting styles and maternal self-efficacy and their association with social-emotional adjustment among Arab preschool children living in Israel. *Parenting Styles Questionnaire*, *Maternal Self-Efficacy Questionnaire*, and *Adjustment Questionnaire* were administered to 420 Arabic-speaking mothers of 3- to 4-year-old children. After employing multiple regression analyses, the results indicated that parenting styles and the overall adjustment of children were significantly correlated. More precisely, a significant association between authoritative parenting style and higher levels of social-emotional adjustment among preschool children was found. Furthermore, maternal self-efficacy was significantly correlated to the overall adjustment of children. In this regard, higher maternal self-efficacy is associated with increased social-emotional adjustment among preschool children. The findings of our study show the applicability of these constructs found relevant across numerous cultures in a unique sample of Arab children living in Israel. Lastly, this study supports intervention programs that promote authoritative parenting style and parental self-efficacy in Arab communities.

## Introduction

Early childhood is a critical period for social-emotional development, which is defined as the development of diverse skills in navigating relationships and coping with an array of emotional states that foster future maturation and psychological health (Izard, 2001). Broadly, as social-emotional competencies flourish, children are presented with opportunities to explore the world around them, face new challenges, and test their ability to cope with adversity. Furthermore, social-emotional competencies are vital for social functioning, helping preschoolers gain the necessary confidence and skills to engage in successful social interactions, including establishing and maintaining positive peer relationships and friendships (Denham and Brown, 2010). These social and emotional competencies and associations with psychological well-being are identified by many scholars as “social-emotional adjustment” which is a gradual, integrative process through which children acquire the capacity to understand, experience, express, and manage emotions and to develop meaningful relationships with others (Aunola et al., 2000; Gülay & Önder, 2013; Santrock, 2004; Yeh, 2003). Social-emotional adjustment is typically evaluated across three domains: interpersonal, emotional, and academic. The interpersonal emotional adjustment relates to one’s ability to interact with other children and his/her social role; the emotional adjustment relates to self-confidence and self-control, while academic emotional adjustment relates to the completion of educational tasks (Kandır & Alpan, 2008).

The development of adequate social-emotional adjustment is especially relevant to children who belong to a minority group that is living in a society where their racial, cultural, or linguistic backgrounds are underrepresented. One group that may have particularly high levels of cultural tension is Arab children living in Israel. This group has to navigate the polarization of the Palestinian versus Israeli national narratives and social demands, which may possibly interfere with social-emotional adjustment (Agbaria et al., 2021; Agbaria, 2020; Dwairy, 2009).

Many studies demonstrate that children who live in multicultural societies have higher levels of open-mindedness, cultural empathy, perceived self-efficacy, and problem-solving strategies (Maddux et al., 2021). However, children who belong to disadvantaged minority groups are often found to display lower levels of emotional stability (Dewaele & Oudenhoven, 2009; Schachner, et al., 2016; Yeh, 2003). Given the distinct situation of Arab children in Israel living in a multicultural social but as a minority group, it is unknown whether their cultural background may contribute to positive or negative outcomes in social-emotional adjustment.

Social-emotional adjustment starts with the child’s social interactions during the first year of life. Parents–child relationship plays a key role in that adjustment from an attachment perspective. It has been proposed that children who develop secure attachments with parents will also develop more positive teacher–child relationships, which may increase their classroom psychosocial adjustment (Breeman et al., 2015). On the other hand, children with low level of social-emotional adjustment may give up more easily on challenging tasks, fail to acquire the skills that develop with persistence, and develop problematic behaviors when faced with subsequent difficult situations.

### Social-emotional adjustment and parenting styles

Parents have major influences on the lives of their young children; they are responsible for modeling culture- and family-specific attitudes, behaviors, and values (McGillicuddy-De Lisi and Lisi , 2007). Parents differ in their methods of sharing and reinforcing these values with their children, and that is what is referred to as parenting styles. Parenting style is classified as authoritarian, authoritative, permissive (Baumrind, 1991), and uninvolved (Veronese et al., 2022). Authoritarian parenting style describes parents who shape and control the behaviors of their children according to clear standards (Zupancic et al., 2004). These parents tend to exhibit reduced affection or warmth and offer minimal encouragement or comfort and minimal to no support to children’s independence/autonomy (Briggs-Gowan et al., 2001). Consequently, children of parents with an authoritarian parenting style have been found to exhibit low self-esteem (Mantzicopoulos & Oh-Hwang, 1998), lower levels of achievement at school (Nyarko, 2011), high levels of depression and hostility, and inability to solve problems on their own (Aunola, et al., 2000; Finzi-Dottan et al., 2011).

Authoritative parenting style combines high levels of control with high levels of support. Thus, the child is both supervised closely and cared for consistently. These parents often take into consideration the personal opinions and desires of their children, offer them explanations for their parenting decisions, and permit negotiations between all members of the household (Weiss & Schwarz, 1996; Zupancic et al., 2004). Children of parents with an authoritative style have not only been found to be more successful, have more friends, and exhibit high levels of self-confidence, but they are also able to complete assigned tasks efficiently and control fluctuations in their emotions (Denham et al., 2000; Mahamid et al., 2022).

Permissive parenting style is characterized by low parental control and high emotional support for the child. In this parenting style, the wishes and actions of the children are prioritized, and the children are not punished when they do not comply with the guidelines (Rossman & Rea, 2005; Zupancic et al., 2004). Children of permissive parents are more likely to rebel, act impulsively, be less successful in reaching objectives, be dependent on others, and act aggressively (Wu, 2009). According to Baumrind (1991), this parenting style is associated with children having diminished independence, increased school absenteeism, and greater involvement in criminal activities. These children have also been found to display a lack of self-control, low social skills, difficulty dealing with autonomy, a lack of maturity, low self-esteem, and feelings of alienation (Abu Baker et al., 2021; Santrock, 2004).

Lastly, uninvolved parents uphold few rules and are emotionally unresponsive (Sanders, 2003). In this parenting style, the complete lack of boundaries in the household makes it difficult for children to learn the appropriate behaviors that can, for example, be applied in schools. Prior findings demonstrate that children with uninvolved parents are more likely to misbehave in social settings (Gimenez-Serrano et al., 2022). Gharrah (2015) suggested that uninvolved parenting style may have the most negative effect on children compared to the other three parental styles.

### Cultural influences on parenting style outcomes

Authoritative parenting style was found to be related to fewer behavioral problems among Arab preschool children in Israel (Agbaria, 2020), though it is unknown how this relates to social-emotional adjustment. This shows that authoritative parenting style affects children similarly across different cultures and ethnicities. Consistent with prior findings in Western societies, recent studies in Arab communities also found that mothers who perceive themselves as permissive or uninvolved assessed their children’s social skills negatively (Abu-Taleb, 2013; Haj-Yahia and Greenbaum, 2021). Relatedly, among a sample of Arab preschool children, permissive and uninvolved parenting styles were associated with greater behavioral problems (Abu Baker et al., 2021). Thus, permissive and uninvolved parenting styles have also been more consistently associated with negative outcomes across cultures.

Interestingly, however, the views around authoritarian parenting style differ cross-culturally. For instance, past research claimed that children from collectivist cultures such as Chinese, African-Americans, and Arabs have long perceived authoritarian parenting style as an expression of care, love, respect, and protection. Therefore, in these traditional cultures, authoritarian parenting style has not been found to be related to psychological distress but rather to improved social-emotional adjustment (Chao, 1994; Dwairy, et al., 2006; Kagitcibasi, 2005). More recent research on parenting styles among Arabs, however, demonstrates that authoritarian parenting style has been associated with poorer social-emotional adjustment (Abu-Taleb, 2013) and behavioral problems (e.g., disruptive classroom behavior) among preschool children (Agbaria, 2020). These contemporary studies show that authoritarian parenting style affects children negatively rather than positively as claimed in previous studies.

Some of the cultural differences that may nuance these findings are highlighted by the theoretical framework developed by Keller (2016) who reported that collectivist societies like traditional Arab cultures typically embody a multiple caretaking model, and thus, examining the role of parenting style requires a nuanced consideration of the parenting arrangement. Specifically, the Arab families living in Israel are faced with dual influences of traditional collectivist Arab culture and the more individualistic, modern values of Israel. Thus, the associations between parenting style with children’s social-emotional adjustment within this unique population warrant examination.

Overall, authoritative parenting style has been consistently associated with positive developmental outcomes across Western and non-Western cultures, including Arab individuals living in Israel. Furthermore, permissive and uninvolved parenting styles have been related to negative outcomes across cultures. However, cultural influences differentially inform the relationships between authoritarian parenting and developmental outcomes, such that collectivist societies (e.g., Arab society) have observed both positive and negative influences. Yet, the interplay between parenting style and social-emotional adjustment has never been explored among Arab individuals living in Israel, which is one gap in the literature that will be addressed in the present work.

### Social-emotional adjustment and maternal self-efficacy

Another factor that has been historically associated with social-emotional adjustment is parental self-efficacy. Parental self-efficacy is the extent to which parents perceive themselves as capable of performing various tasks connected with the role of being a parent. Prior studies showed that parenting characterized by high self-efficacy has been associated with greater social-emotional adjustment among children (Albanese et al., 2019; Li et al., 2010; Steca et al., 2010). A parent with high self-efficacy has high levels of self-confidence in his/her ability to act as a parent, is willing to invest in this task, and believes that he/she has the ability to positively affect the development and behavior of his/her child in a way that meets the child’s needs (Jones & Prinz, 2005; Pelletier & Brent, 2002). Therefore, higher parental self-efficacy is associated with higher quality of parental emotional support and more positive parenting strategies ( Dwairy, 2002; MacPhee and Meller-Heyl, 2003). Mothers who view themselves as having high self-efficacy are more likely to demonstrate a warm and responsive style of parenting combined with strict discipline and control over the child’s behavior, which is consistent with authoritative parenting style (Albanese et al., 2019; Baumrind, 1991). Notably, maternal self-efficacy has been linked to child adjustment in various areas including academic achievement (Jones & Prinz, 2005; Pelletier & Brent, 2002).

Preliminary studies that examined parental self-efficacy among Arab parents found that higher parental self-efficacy led to more positive outcomes among children. Khoury-Kassabri et al. (2013), for example, found that Arab mothers who reported low levels of parental self-efficacy were more likely to report social-emotional maladjustment among their children than mothers who reported higher levels of parental self-efficacy. Moreover, Fass et al. (2017) found that Arab mothers with low maternal self-efficacy had a greater likelihood to punish their children, which in return was associated with lower levels of social-emotional adjustment among children. This supports the notion that higher maternal self-efficacy leading to positive outcomes across different cultures and socioeconomic status, though the relationship with parenting style during the sensitive developmental period of preschool, is unknown and warrants empirical investigation.

### The current study

The primary goal of the current study was to assess whether the associations that have been established cross-culturally between parenting style and maternal self-efficacy with social-emotional adjustment may similarly be applicable within the unique population of Arab preschool children living in Israel who deal with unique challenges which may impact their ability to attain personal and social adjustment and develop a stable identity. Based on previous studies from different cultures (Albanese et al., 2019; Li et al., 2010;  McGillicuddy-De Lisi and Lisi, 2007), this study hypothesized that (1) authoritative parenting style would be associated with higher levels of social-emotional adjustment, compared to authoritarian, permissive, and uninvolved parenting styles, and (2) greater maternal self-efficacy would be associated with higher levels of social-emotional adjustment among children. In order to examine all these variables, this study used reports from teachers about each child’s emotional and psychological adjustment in order to provide a more comprehensive picture of the children’s social-emotional functioning. The findings will be discussed with respect to the unique cultural aspects of Arab preschool children living in Israel.

## Methods

### Participants

The study sample was drawn from 16 government preschools, and the sample size for this study was calculated based on 95% CI and 5% margin of error by using the Raosoft software sample size calculator. Based on that, the recommended sample was 430 Arabic-speaking mothers of 3- to 4-year-old children from the Arab population, within the central part of Israel. We excluded ten questionnaires from the analysis due to inaccurate and incomplete responses. The recruitment of the participants was completed through a convenience sampling method from sixteen public preschools that were chosen from the Ministry of Education districts. Participants from these schools were recruited through online and in-person methods, including social media advertisements and presenting written materials at school meetings or sending them home with children. The mothers’ average age was 30.12 years (*SD* = 9.34). A total of 45.9% of the participants had obtained higher diplomas, 43.2% had obtained university degrees, 8.2% held a high school degree, and 2.7% had only completed primary education. The majority of the participants were married (99.3%), while the other (0.7%) were divorced. In addition to that, each child’s teacher was also surveyed to report on the child’s adjustment in school.

Approximately one-third (33.1%) of the participants had three children, and the second third (32.5%) of the participants had two children, while 28% of the participants had more than three children, and the remaining 6% had only one child. With respect to the children, 50% of them were boys, and 50% were girls. Approximately one-third (33.8%) of the children were the first born, while 38.4% of them were the youngest in the family. A total of 23.2% of the children were the middle child in the family, and the remaining 4.6% were the only child in the family. The children’s average age was 4.12 (*SD* = 1.11). Regarding socioeconomic status, 30.2% of children were from families with high socioeconomic status, 45.7% from families with moderate socioeconomic status, and 23.1% from families with low socioeconomic status. Criteria for including parents and children in the study were as follows: (1) having never been diagnosed with any form of neurodevelopmental or psychological impairment, (2) living in Israel, and (3) attending public preschools in Israel. The teachers included in the study were all working within public preschools in Israel.

### Measures

Following standard methodological recommendations for developing our questionnaires, all items were translated and back-translated from the original English version to Arabic and pilot tested by a panel of ten Arab professionals recognized as experts in psychology, counselling, and social work. These professionals evaluated the clarity and relevance of the questions and translation. After completing the translated draft, an independent expert English editor back-translated the questionnaires into English. The translated version was then pilot tested among 70 participants (validity sample) and further refined for clarity.

#### Personal information questionnaire

The mothers self-reported on the following questions: mother’s age, mother’s education level, family status (married/divorced), child’s age, child’s gender, child’s birth order, child’s socioeconomic status, and number of children in family.

#### Parenting styles questionnaire

This is a 49-item self-report measure developed by Abu Taleb (2013) in Arabic to categorize respondents’ parenting styles based on the four categorizations: 14 items for authoritative, for example: “There is a strong connection between myself and my child”; 12 items for authoritarian, for example: “I am always in control of my child”; 10 items for permissive, for example: “No limits are placed on the child in the home”; and 12 items for uninvolved, for example: “Do not respond quickly to the needs of the child.” This questionnaire was selected because it was developed specifically for use in Arab populations. Each respondent receives an average score for each of the four parenting styles, ranging from 1 (low) to 3 (high). The subscales of the measure have demonstrated good internal consistency (authoritative: *α* = 0.82; authoritarian: *α* = 0.79; permissive: *α* = 0.70; uninvolved: *α* = 0.80) (Abu-Taleb, 2013). In the current study, good internal consistency was also observed: (authoritative: *α* = 0.73; authoritarian: *α* = 0.74; permissive: *α* = 0.70; uninvolved: *α* = 0.69).

#### Maternal self-efficacy questionnaire

The original questionnaire contains 30 self-report items (Aviram, 1990) and measures the individual’s expectations regarding their ability to initiate and follow through with effective behavior across various domains of one’s personality, for example, “I feel insecure as to my ability to get things done.” Responses to each item were recorded on a 4-point Likert scale ranging from 1 (highly disagree) to 4 (highly agree), and an average score was calculated. The questionnaire demonstrated a good internal consistency (*α* = 0.83). In our study, we used the modified version of this questionnaire reflecting self-efficacy in parenting practices which was used by Keller (2012). The questionnaire exhibited good internal consistency (*α* = 0.86), which was similarly observed in the current sample (*α* = 0.72).

#### Adjustment questionnaire

Child adjustment was measured using the “Adjustment scale of children to kindergarten and school for teachers” (Smilansky & Shfatyah, 2001). The scale includes 18 questions to measure adjustment in three areas: academic (e.g., ability to meet deadlines), emotional (e.g., expressing feelings appropriately), and social (e.g., maintaining peer relationships). Each question describes a facet of the child’s behavior evaluated on a 5-point Likert scale from 1 (highly disagree) to 5 (highly agree). The scale has exhibited good internal consistency for the composite scores of all items, general adjustment (*α* = 0.88), and for the subscales: academic adjustment (*α* = 0.66), emotional adjustment (*α* = 0.77), and social adjustment (*α* = 0.68). The measures of internal consistency reported by Bouley (2011) for the “Adjustment Assessment Scale” were *α* = 0.91 for the entire scale and *α* = 0.77 and 0.88 for social and emotional adjustment, respectively. For the current study, the measures of reliability were also good for general adjustment (across all three domains) (*α* = 0.94), emotional adjustment (*α* = 0.82), and social adjustment (*α* = 0.84).

### Procedures

The study was carried out in sixteen public preschools in Israel during the 2020–2021 school years. The research team first received approval from the government’s chief scientist and the Institutional Review Board (IRB) of An-Najah National University. Participants from these sixteen schools were recruited from online advertisements, e-mail campaigns, and social media. The aims and procedures of the study were explained online, and parents who were interested in participating were sent an email clarifying their willingness to participate in the study. Each child attending the selected sixteen public preschools received a letter at school to give to their parents. The letter briefly explained the subject of the study and its purpose, including ethical issues of confidentiality and voluntary participation. Informed consent was obtained from all individual participants included in the study, including both the mothers and teachers. Questionnaires were distributed to the participating mothers and the children’s preschool teachers with instructions on how to complete the measures at home. Nearly all of individuals who consented to participate in the study completed the questionnaires (95%). Sixteen teachers completed the adjustment questionnaire for children. Coded matching procedures maintained the coordination and anonymity of the gathered data.

### Data analytic plan

Bivariate, zero-order correlations were examined for the study variables to test the associations of parenting styles and maternal self-efficacy with social-emotional adjustment. This allowed for the determination of which variables were significantly associated with social-emotional adjustment and warranted inclusion within a stepwise multiple regression model. Next, the stepwise multiple regression model, with parenting styles subscales and maternal self-efficacy total score significantly associated with social-emotional adjustment, was entered as independent variables and was created to examine the unique associations of each parenting styles and maternal self-efficacy with children’s social-emotional adjustment. Entering all of the significant independent variables into a single regression model allows for the isolation of the effect of each variable while reducing multi-collinearity concerns. In this model, demographic variables for the child’s age, gender, and socioeconomic status were entered in step 1 in order to control for the possible confounding effects of individual differences when examining associations between the study variables in step 2.

## Results

Table 1 depicts the descriptive statistics for parenting styles, maternal self-efficacy, and social-emotional adjustment.

**Table 1:** Descriptive statistics of study variables (*n* = 420)

	**Mean**	***SD***	**Min**	**Max**
Self-efficacy	3.19	0.66	1.18	4.00
Permissive parenting style	1.77	0.58	1.22	3.00
Uninvolved parenting style	2.96	0.83	1.22	3.00
Authoritative parenting style	1.36	0.24	1.00	2.29
Authoritarian parenting style	2.37	0.31	1.38	2.85
General adjustment	3.72	0.80	1.72	5.00
Emotional adjustment	3.91	0.79	1.67	5.00
Social adjustment	3.64	0.86	1.50	5.00

The first hypothesis focused on the association between the child’s adjustment and parenting styles. In support, Table 2 shows a significant, positive correlation between authoritative parenting style and the child’s overall adjustment (*r* = 0.51, *r*^2^ = 0.26, *p* < 0.01) as well as social (*r* = 0.49, *r*^*2*^ = 0.24, *p* < 0.01) and emotional adjustment (*r* = 0.49, *r*^*2*^ = 0.24, *p* < 0.001). There were also significant, negative correlations between authoritarian parenting style and the child’s overall adjustment (*r* =  − 0.39, *r*^2^ =  − 0.78, *p* < 0.01), social adjustment (*r* =  − 0.39, *r*^2^ =  − 0.78, *p* < 0.01), and emotional adjustment (*r* =  − 0.37, *r*^2^ =  − 0.74, *p* < 0.01). Furthermore, there were significant, negative correlations between uninvolved parenting style and the child’s overall adjustment (*r* =  − 0.30, *r*^3^ =  − 0.60, *p* < 0.01), social adjustment (*r* =  − 0.33, *r*^2^ =  − 0.66, *p* < 0.01), and emotional adjustment (*r* =  − 0.40, *r*^*2*^ =  − 0.80, *p* < 0.01). No associations were observed between permissive parenting style and any facets of adjustment (*p*s > 0.05).

**Table 2:** Correlations of study variables (*n* = 420)

	1	2	3	4	5	6	7	8
1. Self-efficacy		-	-	-	-	-	-	-
2. General adjustment	0.38**		-	-	-	-	-	-
3. Emotional adjustment	0.30**	0.91**		-	-	-	-	-
4. Social adjustment	0.34**	0.94**	0.82**	-	-	-	-	-
5. Permissive style	0.13	− 0.12	− 0.13	− 0.11	-	-	-	-
6. Uninvolved style	− 0.22**	− 0.30**	− 0.33*	− 0.40**	0.27**	-	-	-
7. Authoritative style	0.34**	0.51**	0.49*	0.49**	.01	− 0.22**	-	-
8. Authoritarian style	− 0.33**	− 0.39**	− 0.37*	− 0.39**	0.10	− 0.52**	− 0.32**	-

^*^p < .05; **p < .01

Table 3 shows the associations observed in the regression analyses specifying facets of adjustment as the outcome variables and entering all other study variables to isolate the unique effect of each independent variable. Consistent with the correlational analyses, authoritative parenting positively and significantly associated with general adjustment at (*B* = 1.31, *p* < 0.01), social adjustment (*B* = 1.13, *p* < 0.01), and emotional adjustment (*B* = 1.40, *p* < 0.01). Furthermore, authoritarian parenting style exhibited a significant negative contribution in explaining the variance in general adjustment (*B* =  − 0.50, *p* < 0.01), social adjustment (*B* =  − 0.62, *p* < 0.01), and emotional adjustment (*B* =  − 0.63, *p* < 0.01). In addition, uninvolved parenting style also provided a significant negative contribution to the variance for general adjustment (*B* =  − 1.11, *p* < 0.01), social adjustment (*B* =  − 1.18, *p* < 0.01), and emotional adjustment (*B* =  − 1.31, *p* < 0.01).

**Table 3:** Regression analysis for predicting adjustment (*n* = 420)

	General adjustment		Social adjustment		Emotional adjustment	
	*R*^2^	*Β*	*R*^2^	*Β*	*R*^2^	*Β*
**Step 1**	.03		.02		.03	
Child’s age		.06		.05		.06
Child’s gender		.03		.04		.03
Child’s socioeconomic status		.04		.03		.03
**Step 2**	0.26		0.27		0.27	
Uninvolved parenting style		−1.11**		−1.18**		−1.31**
Authoritative style		1.31**		1.13**		1.40**
Authoritarian parenting style		−0.50**		−0.62**		−0.63**
Self-efficacy		0.30**		0.28**		0.29**

**p < .01

The second hypothesis focused on the association between child’s adjustment and maternal self-efficacy. Consistent with hypotheses, significant positive correlations were found between maternal self-efficacy and child’s overall adjustment (*r* = 0.38, *r*^*2*^ = *p* < 0.01), social adjustment (*r* = 0.30, *r*^*2*^ = 0.09, *p* < 0.01), and emotional adjustment (*r* = 0.29, *r*^*2*^ = 0.08, = , *p* < 0.01). In regression analysis, self-efficacy positively and significantly predicts variance in general adjustment (*B* = 0.30, *p* < 0.01), social adjustment (*B* = 0.28, *p* < 0.01), and emotional adjustment (*B* = 0.29, *p* < 0.01).

## Discussion

This current study examined the relationship between parenting styles, maternal self-efficacy, and social-emotional adjustment among Arab preschool children. Consistent with the study hypotheses, the results show that higher overall, social-emotional adjustment among Arab preschool children was associated with authoritative parenting style and higher maternal self-efficacy. Furthermore, lower overall and social and emotional adjustment were found to be related to authoritarian and uninvolved parenting styles.

### Social-emotional adjustment and parenting styles

#### Authoritative parenting style

The findings of this study are consistent with results from prior research, which found that preschool children of parents with an authoritative style have higher social-emotional adjustment than those with other parenting styles (e.g., Albanese et al., 2019; McKinny et al., 2008). Children with authoritative parents exhibit lower levels of stress and depression as they are more likely to feel happy, respected, and appreciated by their parents (Baumrind, 1971; Alegre, 2010). These factors have been specifically related to higher social-emotional adjustment in previous studies and may also be contributing to the current findings (Agbaria, 2014; Alegre, 2010). Furthermore, some of the specific techniques of authoritative parenting style, such as promoting appropriate expectations for children while being sensitive to their emotional experience, may foster more advanced social-emotional skills among them (Webster-Stratton et al., 2011).

#### Authoritarian style

The negative association between authoritarian parenting style and preschool children’s social-emotional adjustment is congruent with prior studies that have demonstrated a negative association between authoritarian parenting style and social-emotional maladjustment during childhood and adolescence (Abu-Taleb, 2013; Baumrind, 1991). Authoritarian parents have a restrictive style of interaction with their children that may discount the child’s opinions in a manner that has been associated with anxiety, fear, and frustration among children (Chen and Wang, 2011). Moreover, children of authoritarian parents have not only been shown to be less content and secure but also more likely to become hostile and have greater difficulties navigating stressful circumstances (Eisenberg et al., 1997; Weiss and Schwarz, 1996).

Interestingly, the current findings suggest that the authoritarian parenting style has negative effects on the social-emotional adjustment of Arab preschool children living in Israel. This contradicts earlier findings that entail that authoritarian parenting style is an ideal approach for children’s development in non-Euro-American ethnic communities (Chao, 1994). The present findings may be related to the process of modernization that has been occurring in Israel over the last decade. Dwairy (2004) noted that the authoritarian parenting style was the most common parenting style among the Arab population living in Israel. However, more recent studies show that authoritative, rather than authoritarian, is the most preferable parenting style for this population (Agbaria et al., 2021).

#### Uninvolved parenting style

Akin to authoritarian parenting style, there was a negative association between uninvolved parenting style and child’s social-emotional adjustment. This result is consistent with prior studies (Abu-Taleb, 2013; Baumrind, 1991). Uninvolved parents are considered “hands-off” in terms of both rule setting and emotional responsiveness, thus contributing to the child’s social and emotional maladjustment. This style has also been associated with poorer academic performance which may similarly contribute to increased social and emotional maladjustment problems (Dwairy and Achoui, 2006). Uninvolved parenting style demonstrates negative behavioral modeling for children, as it may lead the child to feelings of being unwanted and neglected. Thus, these children may act out for attention in social situations or develop poor strategies for emotion regulation because they are not able to learn appropriate behaviors from their parents.

The negative impacts of uninvolved parenting style that have been observed cross-culturally in prior studies also extend to this unique sample of Arab individuals living in Israel. Nevertheless, the participants within the current study actually aligned with the uninvolved parenting style to a greater degree than any of the other four parenting styles. This raises a particular concern that Arab parents living in Israel may be adopting the uninvolved parenting style due to living in an at-risk community and being faced with numerous demands that distract their resources away from more involved parenting styles. Further research is needed to better understand parents’ rationales for adopting the uninvolved parenting style within this population and raise awareness into its negative effects.

#### Permissive style

The current findings paralleled prior research, which did not observe a significant association between permissive style and children’s social-emotional adjustment (Haj-Yahia and Greenbaum, 2021). Yet, the broader picture of this relationship remains inconclusive, as other studies have either observed a positive relationship between the two variables (Chen and Wang, 2011; Hughes and Gottlieb, 2004) or a negative relationship ( Gimenez-Serrano et al., 2022; Wolfradt et al., 2003). These inconclusive results may be explained according to the children’s individual differences regarding how they respond to having fewer boundaries. While some children may develop problems in social and emotional skills (Milevsky et al., 2007), some of them may seek other role models or even develop more mature skills to provide themselves with the structure that their parents were not able to provide. Thus, future research should explore individual factors that may determine which children benefit or suffer from permissive parenting within this unique sample of Arab preschool children living in Israel.

The results of our study could be interpreted from a developmental perspective, the child’s personality and his growth as an adult depends extensively on how he was treated by his parents (Albanese et al., 2019; Li et al., 2010). The education of children is not a spontaneous nor a random action; parenthood is a mixture of joy and struggle for everyone, and no one can claim to hold all the answers to the problems of raising a child. Every parent has his identity and uniqueness. As every family is unique, positive parenting skills are not innate; they are learned (Li et al., 2010). The parents–children relations are healthy when they are oriented in order to take care and not to control or manipulate. A child learns by and through interaction with others (adults, peers, etc.). Thus, quite frequently, we can remark the consequences of a permissive attitude of some parents; they find it beneficial for a child to develop in his own rhythm. A child who grows up without rules does not have references; a child who gets everything he wants without making any effort will be disgruntled and with no motivation. On the other hand, there are the authoritarian parents who make decisions for the child, require obedience of rules, and define firmly the space and its manifestation. Mistakes made in meeting child’s needs can hinder his development.

### Social-emotional adjustment and maternal self-efficacy

Maternal self-efficacy was associated with greater social-emotional adjustment among Arab preschool children in Israel, which is consistent with prior studies in broader demographic samples (Jones & Prinz, 2005; MacPhee & Meller-Heyl, 2003). The confidence of a mother with high self-efficacy may be instilled into her child when approaching new situations, as the mother would feel more comfortable teaching the child the necessary skills and providing warm support. Sharing ideas between mother and child has also been found to encourage the child to share ideas with his/her preschool teacher, which then strengthens the teacher–child dyadic relationship and fosters the child’s adjustment to preschool (Bouley, 2011). Furthermore, a child who has a close relationship with his/her mother may also feel secure in establishing new relationships with children from different ages as he/she is better able to integrate socially (Howes et al., 1994). This would ultimately contribute to higher social-emotional adjustment among preschool children.

Maternal self-efficacy may be particularly important for children’s social-emotional adjustment within the current sample, because Arab children in Israel are living in what is considered a “risk environment,” whereby parents feel that the community will not protect their children. This environment pushes parents to take the lead in promoting their children and protecting them from the negative effects of their surroundings. Political instability, combined with high rates of unemployment and increased poverty, may reduce the levels of self-efficacy among Arab parents in Israel. This shows the need to promote parental self-efficacy within this sample as higher parental self-efficacy may help the child become better equipped to confidently navigate such risk environments.

These results show that while culture plays an important role in mediating the relationship between parenting styles, parental self-efficacy, and child’s social-emotional development, it is also important for future research to take into account the socioeconomic status of the families under study.

There are several practical implications that may be speculated based on the preliminary, cross-sectional results of this research. For instance, the association of authoritative parenting style with higher social-emotional adjustment in children may inform educational efforts and intervention programs for Arab parents. Also, individuals with regular contact with the parents (e.g., the child’s primary care physician, the child’s teacher, etc.) may consider advocating for the importance of parental strategies consistent with the authoritative parenting style (e.g., consistent expectations and high warmth). These educational efforts could also be extended to the training of educators by having each teacher take a course to develop their knowledge and expertise regarding the social and emotional adjustment of children that emphasizes the principles of authoritative parenting, which can also be replicated by the teachers themselves (e.g., firm behavioral standards with high support).

Given the association between maternal self-efficacy with higher levels of children’s social-emotional adjustment, intervention efforts could also be targeted towards improving maternal self-efficacy. It would be especially beneficial for expecting mothers to foster their parental self-efficacy by setting appropriate expectations and thinking through possible challenges during the prenatal period. This early intervention could take place at parenting and family centers and be facilitated by experts on the subject. Thus, the present, cross-sectional findings provide initial insight into how maternal self-efficacy and parenting style may warrant consideration in clinical applications, though future research including longitudinal approaches is needed to better understand the most relevant intervention strategies. We also recommend testing the variables of the current study using structural equation modeling (SEM) to identify predictor, mediating, and outcome variables.

### Limitations

The current study contained several key limitations. This sample was intentionally recruited from mothers of preschool children in a defined geographic area and linguistic/religious subgroup that was not random. Therefore, the findings may not be generalizable to different demographic groups. The study also relied on self-report for both parenting style and maternal self-efficacy which may be limited by the respondent’s potential bias. Future studies can benefit from adding behavioral indices or external evaluations and assessing the child’s socio-emotional ability directly. This would allow for further insights regarding the social and emotional development of children. Relatedly, in the current study, parents did not provide ratings on their child’s adjustment (only the teachers did), which should be considered in later work to get a more comprehensive picture of childhood adjustment across multiple settings (e.g., school and home). In addition, future research may also consider sampling fathers, in addition to mothers, as previous studies have found that the parenting styles of the fathers and mothers may differ from one another (McGillicuddy-De Lisi & Lisi, 2007). Thus, surveying both mothers and fathers may provide a more comprehensive picture of the effects that parenting styles and self-efficacy have on children’s social-emotional adjustment. The scales used in our study and their psychometric characteristics had not previously been tested with this specific population, and so atypical results cannot be fully ruled out. Lastly, further research in this area, or with this population of Arab preschool children living in Israel, could also provide a more comprehensive picture by assessing how demographic variables (e.g., how many children live in the home, birth order), positive behavioral outcomes (social-emotional adjustment), and negative behavioral outcomes (e.g., aggression) associated with parenting styles and parental self-efficacy.

## Conclusion

In summary, the present study provided support that associations between social-emotional functioning in youths with parenting style and maternal self-efficacy observed in numerous cultures are also applicable within this unique sample of Arab preschool children living in Israel. Specifically, higher social-emotional adjustment among Arab preschool children was associated with authoritative parenting style and higher maternal self-efficacy. In contrast, authoritarian and uninvolved parenting styles, as well as lower maternal self-efficacy, were associated with poorer social-emotional adjustment among the sampled children. While these findings attest that authoritative parenting style and higher maternal self-efficacy are important for the child’s development cross-culturally, future research might also consider examining the role of the family’s socioeconomic status in mediating the relationship between parenting styles, parental self-efficacy, and child’s adjustment. Promoting authoritative parenting style and increasing parental self-efficacy may be especially important for Arab parents and children living in Israel as they are considered an at-risk population. Thus, additional support may be necessary for parents from this population to successfully foster their children’s social-emotional adjustment.

## Data Availability

The datasets generated during and/or analyzed during the current study are available from the corresponding author on reasonable request.

## Abbreviations

IRB: Institutional review board
SEM: Structural equation modeling
